# PINK1, cancer and neurodegeneration

**DOI:** 10.18632/oncoscience.284

**Published:** 2016-01-12

**Authors:** Ciara H. O'Flanagan, Vanessa A. Morais, Cora O'Neill

**Affiliations:** School of Biochemistry and Cell Biology, BioSciences Institute, University College Cork, Cork, Ireland

**Keywords:** Parkinson disease, mitochondrial dynamics, cell cycle checkpoints, carcinogenesis, mitochondrial degradation

Cancer and neurodegeneration are two age-related diseases that arise from aberrant signaling in similar cellular systems, those that balance survival and death. Thus, deregulated molecular processes such as DNA damage repair, intracellular energy balance, and key signal transduction systems, including the PI3-kinase/Akt axis can promote tumorigenesis and induce neurodegeneration [1]. Epidemiological studies support this cross-talk between cancer and neurodegeneration, indicating a reduced risk of certain cancers in patients diagnosed with neurodegenerative diseases such as Parkinson's disease (PD) [2]. In addition, several of the genes discovered to cause inherited PD, including PTEN induced putative kinase 1 (PINK1) have been described to have oncogenic or tumor suppressor properties [3].

In a recent study we focused on the function of PINK1 in cancer cell biology, and discovered a novel function for PINK1 as a positive regulator of cell cycle progression that can promote cancer-associated phenotypes [4]. PINK1 is ubiquitously expressed and was named due to induction by the tumor suppressor PTEN in cancer cells, drawing attention to its putative role in cancer from the first instance. Several mechanistic links between PINK1, PTEN and the PI3-kinase/Akt signaling axis that PTEN inhibits were subsequently highlighted, indicating PINK1 is both regulated by and regulates PI3-kinase/Akt signaling [5]. Interlinked with this, in an as yet undefined manner, PINK1 is best described as a major mitochondrial quality control protein, rudimentary to cell survival due to its regulatory role in the triad of mitochondrial fission, fusion and mitophagy as well as mitochondrial bioenergetics.

Although somewhat understudied, the cell cycle and mitochondrial quality control are intrinsically coupled [6]. Mitochondria must divide and undergo fission during mitosis to allow equal distribution of mitochondria to daughter cells, also permitting clearance of damaged mitochondria via mitophagy. Conversely, mitochondrial fusion occurs during the transition from mitosis to G1 following cytokinesis, and can promote stress resistance and cell cycle exit in G0. Our findings show for the first time that regulation of mitochondrial fission to fusion transitions by PINK1 is critical for cell cycle progression at G2/M and G0/G1 checkpoints necessary for cell division, growth and stress resistance, in particular in cancer biology. In line with this, PINK1 deletion reduced proliferation, colony formation, migration and invasive potential in several cell model systems.

In further detail, PINK1-deficiency induced multinucleation and cell cycle arrest during G2/M and resulted in a reduced ability to exit the cell cycle following serum withdrawal. This was PINK1 kinase dependent and rescued by re-introduction of human PINK1. The cell cycle changes induced by PINK1 deletion where mechanistically linked to excessive mitochondrial fission, and increased expression and activation of the master mitochondrial fission protein dynamin-related protein 1 (Drp1). siRNA knockdown of Drp1 and restoration of mitochondrial fusion in PINK1-deficient cells caused a reduced multi-nucleation. Together this indicates that mitotic arrest with an inability to complete cytokinesis in cells without PINK1 is due to excessive mitochondrial fission, and an inability to induce fusion following nuclear separation and prior to cytokinesis. Significant cell cycle marker changes were co-existent with this defect including failure to increase cyclin D1, indicative of mitotic arrest induced by PINK1 deletion.

PINK1 has been previously highlighted as a potential target for cancer therapy and been shown to sensitize cancer cells to DNA damaging agents and chemotherapeutic drugs [7]. Our findings show that PINK1 inhibition constrains proliferation, halting the cell just before division, the point at which many of these agents target. PINK1 may therefore be a direct target to block the cell cycle in cancer or for combination therapies to ‘prime’ cancer cells for treatment with other mitosis-targeting drugs. Conversely, the inability of cells to effectively divide in the absence of PINK1 has the potential to increased chromosomal aberrations, genetic instability and aneuploidy that could lead to cancer in some cell types. This context dependent pro- and antitumorigenic properties depending on cell type, is emerging for many genes with oncogenic potential.

The significance of PINK1's involvement in cell cycle regulation is important not only in cancer whereby cells are continually dividing, but also in neuronal biology, as abortive cell cycle re-entry in terminally differentiated, post-mitotic neurons has been suggested to be a key mechanism in neurodegenerative diseases [3]. While much is known about the function of PINK1 in mitochondrial biology and also to a lesser but growing extent, in PI3-kinase/Akt signalling, the exact mechanism through which loss of function of PINK1 causes PD is still unknown. This new function of PINK1 as a regulator of the cell cycle draws attention to the function of PINK1, via mitochondrial quality control, in both cell division, and cell differentiation programs, that underlie cancer and adult neuronal phenotypes. These findings therefore add another piece towards solving the larger puzzle of PINK1 function in neuronal systems and highlight the potential of PINK1 as a target in future anti-cancer therapies.
